# DNA damage and repair capacity in lymphocyte of chronic obstructive pulmonary diseases patients during physical exercise with oxygen supplementation

**DOI:** 10.1186/s40248-016-0079-7

**Published:** 2016-12-14

**Authors:** Andréa Lúcia Gonçalves da Silva, Thaís Evelyn Karnopp, Augusto Ferreira Weber, Cassia da Luz Goulart, Paloma de Borba Scheneiders, Dannuey Machado Cardoso, Lisiane Lisboa Carvalho, Joel Henrique Ellwanger, Lia Gonçalves Possuelo, Andréia Rosane de Moura Valim

**Affiliations:** 1 Departamento de Educação Física e Saúde, Universidade de Santa Cruz do Sul – UNISC, Avenida Independência, 2293, Bairro Universitário, Santa Cruz do Sul, RS CEP 96815-900 Brazil; 2 Hospital Santa Cruz, Santa Cruz do Sul, RS Brazil; 3 Departamento de Biologia e Farmácia, Universidade de Santa Cruz do Sul – UNISC, Santa Cruz do Sul, RS Brazil; 4 Programa de Pós-Graduação em Genética e Biologia Molecular, Departamento de Genética, Universidade Federal do Rio Grande do Sul - UFRGS, Porto Alegre, RS Brazil; 5 Programa de Pós-Graduação em Promoção da Saúde, Universidade de Santa Cruz do Sul – UNISC, Santa Cruz do Sul, RS Brazil

**Keywords:** DNA repair, Aerobic exercise, Comet assay, Induced DNA damage, COPD

## Abstract

**Background:**

We hypothesized that the use of oxygen supplementation during aerobic exercise induces less DNA damage than exercise alone.

The aim of this study is to assess the level of DNA damage induced by physical exercise with and without oxygen supplementation in chronic obstructive pulmonary diseases (COPD) patients.

**Methods:**

Peripheral blood was collected before and after aerobic exercise in two conditions: (I) aerobic exercise without oxygen supplementation (AE group) and (II) with oxygen supplementation (AE-O_2_ group). Lymphocytes were collected to perform the alkaline version of the Comet Assay. To assess the susceptibility to exogenous DNA damage, the lymphocytes were treated with methyl methanesulphonate (MMS) for 1-h or 3-h. After 3-h treatment, the percentage of residual damage was calculated assuming the value of 1-h MMS treatment as 100%.

**Results:**

AE group showed lower induced damage (1 h of MMS treatment) and consequently less DNA repair compared to AE-O_2_ group. AE-O_2_ group showed an increase in the induced DNA damage (1 h of MMS treatment) and an increased DNA repair capacity. Within the AE-O_2_ group, in the post-exercise situation the induced DNA damage after 1 h of MMS treatment was higher (*p* = 0.01) than in the pre-exercise.

**Conclusion:**

COPD patients who performed physical exercise associated with oxygen supplementation had a better response to DNA damage induced by MMS and a better DNA repair when compared to the condition of physical exercise without oxygen supplementation.

**Trial registration:**

UNISC N374.298. Registered 04 JUN 2013 (retrospectively registered).

## Background

Chronic obstructive pulmonary disease (COPD) is a lung disorder with progressive airflow obstruction resulting from inflammation and remodeling of the airways, which often includes the development of emphysema. Although the lung is the primary organ affected by the disease, COPD is increasingly acknowledged as a systemic disease due to its clinically significant extra-pulmonary consequences [1, 2]. Systemic degenerative manifestations in COPD include osteoporosis, muscle wasting, reduced exercise capacity, systemic inflammation, loss of muscle mass, and reduced physical activity [3, 4]. The prevalence of muscle atrophy is relatively high in COPD patients, ranging from 20 to 40% depending on disease stage [5]. The high mortality rate of COPD patients is associated with decreased physical capacity. However, it can be improved through pulmonary rehabilitation [4].

Exercise may improve gas exchange in subjects with mild COPD, largely due to an improvement of the ventilation/perfusion (V/Q) relationship resulting from the more even distribution of ventilation. However, in more severe disease, V/Q mismatching and peripheral oxygen extraction are increased [6], and dynamic hyperinflation contributes to alveolar hypoventilation, with resultant exertional hypoxemia [7]. Studies have shown an increased systemic oxidative stress response after strenuous exercise in patients with COPD [8, 9]. On the other hand, higher concentrations of reactive oxygen species can mediate damage to lipids, proteins, and DNA, and drive inflammatory cascades. Oxidative stress impairs skeletal muscle contractility. COPD patients have increased oxidative stress, particularly following exercise [10, 11]. This effect appears particularly marked in chronically hypoxemic subjects, in whom markers of oxidative stress are significantly increased in peripheral muscle specimens at rest and following exercise. However, supplemental oxygen can reduce pulmonary hypertension and frequency of exacerbations, increasing exercise tolerance [6], thereby releasing more energy for locomotor muscles [12].

Desaturation with exercise appears to predict increased risk of mortality [13]. However, the role of the supplemental oxygen in this scenario is little known. While often considered as an attractive therapeutic option, the use of supplemental oxygen is not free of risk or adverse consequences (DNA damage, for example) [6]. It was shown that supplemental oxygen provides short-term symptom relief and improves exercise performance, but longer-term data are lacking [14]. In this context, we hypothesized that the use of oxygen supplementation during aerobic exercise induces less DNA damage than aerobic exercise alone. Therefore, the aim of this study was to assess the level of DNA damage induced by physical exercise with and without oxygen supplementation in COPD patients.

## Methods

### Subjects and ethical aspects

This crossover study is part of a larger study involving other lung diseases besides COPD, as tuberculosis and lung cancer. Twenty-five COPD patients determined by convenience were included in this study, with an average age of 64.40 ± 7.66 years, treated at the *Hospital Santa Cruz* (Santa Cruz do Sul, Rio Grande do Sul, Brazil) by the Research Group for Health Rehabilitation. At the beginning of the study, all participants underwent pulmonary rehabilitation 3 times per week for 8 weeks (i.e., endurance physical training and education about the disease) [15]. Aerobic exercise in vertical cycle ergometer (30 min per session) was conducted by a physiotherapist, initially at an intensity of 50% of the heart rate, according to Karvonen method and control by Borg Scale of perceived exertion. Participants also performed upper and lower limb strengthening exercises with weights and muscle stretching [15]. COPD patients were diagnosed according to the Global Initiative for Chronic Obstructive Lung Disease Guidelines - GOLD [15], by clinical history, physical examination, and presence of airflow obstruction. All subjects were informed about the study and provided written consent prior to being included in the research.

### Experimental design

**Fig. 1:**
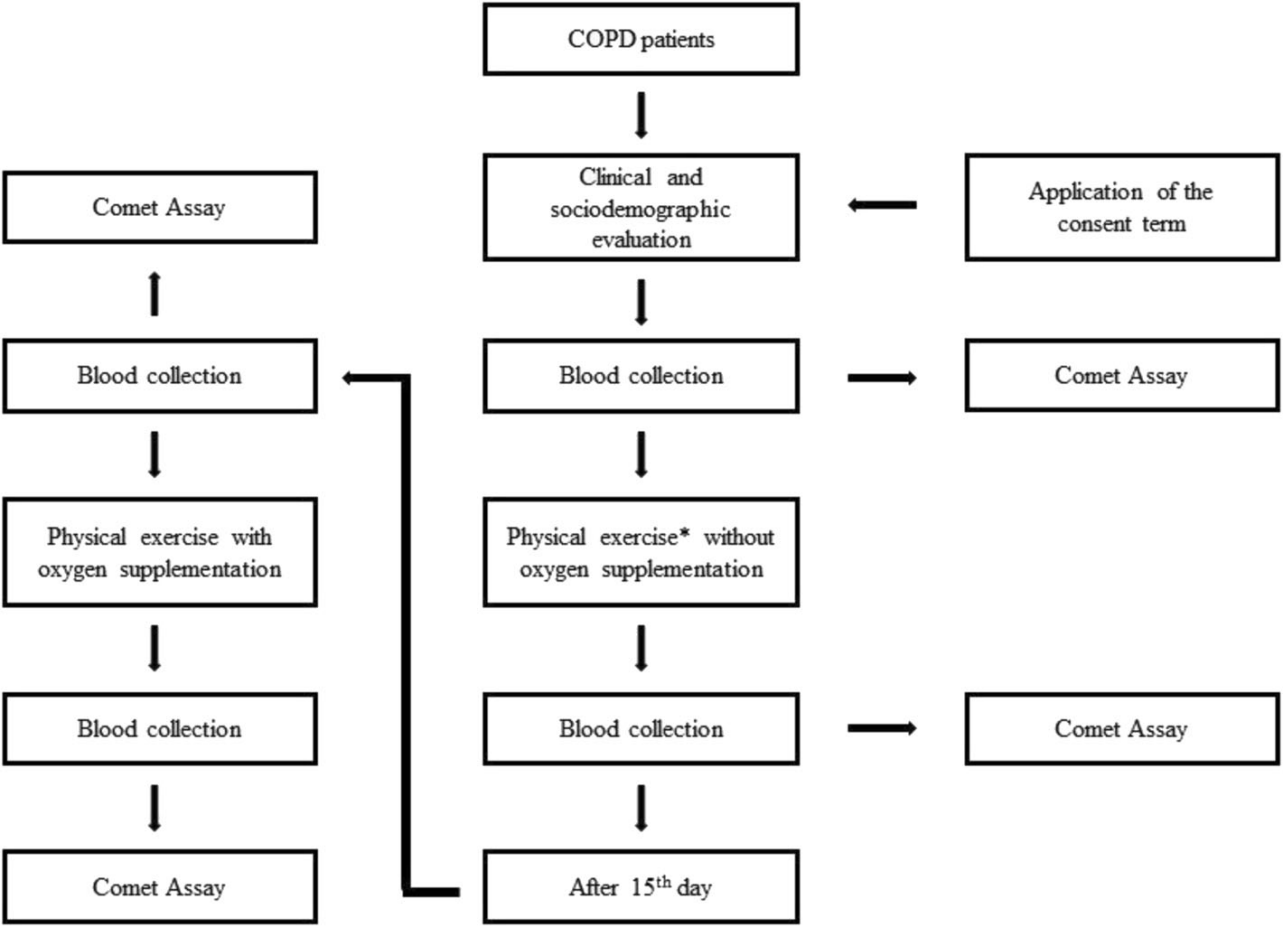
Experimental design of the study. COPD, Chronic obstructive pulmonary disease. *Consisted of aerobic exercise performed during 30 minutes in lower limb cycle ergometer (intense training, up to 50% of the maximum heart rate)

Patients were submitted to blood collection in different moments, with an interval of 15 days between them. The first moment comprised the group of COPD patients in aerobic exercise without O_2_ supplementation (AE group). In the second moment, the same patients performed aerobic exercise with O_2_ supplementation (AE-O_2_ group). The oxygen supplement (FiO_2_) was adjusted to maintain the peripheral oxygen saturation above 90% during exercise. The experimental design of the study is shown in Fig. 1.

### Blood collection

Peripheral blood samples were collected in two moments, before and after aerobic exercise, into two tubes with anticoagulant. One aliquot was used for the extraction of lymphocytes for subsequent Comet Assay and the other aliquot was used to obtain blood plasma for the remaining analysis.

### DNA damage evaluation by Comet Assay

The Comet Assay was performed under alkaline conditions [16, 17]. Aliquots of 20 *μ*L whole lymphocytes were mixed with 200 *μ*L low melting point agarose (0.7% in phosphate buffer) and added to microscope slides precoated with 1.5% agarose. The slides were then incubated in ice-cold lysis solution (2.5 M NaCl, 100 mM EDTA, 10 mM Tris, 20 mM NaOH, pH 10.2, 1% Triton X-100, and 10% DMSO). After 24 h, the slides were removed from the lysis solution and placed in an electrophoresis unit filled with fresh electrophoresis buffer at 4 °C. In the alkaline version of the Comet Assay (10 M NaOH, 1 mM EDTA, and pH >13), 20 min of denaturation and 15-min electrophoresis time were used. For DNA damage evaluation, 100 cells per sample were analyzed by optical microscopy at 100x magnification. The cells were visually scored by measuring the DNA migration length and the amount of DNA in the “tail” into five classes, ranging from undamaged (damage 0) to maximally damaged (damage 4). Then, the damage index (DI) was calculated for each sample, ranged from 0 (completely undamaged: 100 cells × 0) to 400 (with maximum damage: 100 cells × 4) [18].

### DNA damage repair

For the assessment of susceptibility to exogenous DNA damage, whole lymphocytes were treated with the alkylating agent methyl methanesulphonate (MMS) (8 × 10-5 M) for 1 h or 3 h at 37 °C prior to slides preparation. The percentage of residual DNA damage after 3-h MMS treatment was calculated using the value of 1-h MMS treatment for each subject as 100%. MMS is a DNA alkylation agent widely used as positive control in genotoxicity testing [19].

### Statistical analysis

The statistical analyses were performed using the Statistical Package for the Social Sciences 20.0 and *p* < 0.05 was considered statistically significant. Data were expressed as mean and standard deviation or median (interquartile range) for nonparametric data. Wilcoxon test was used to evaluate the data relating to Comet Assay between the pre- and post-exercise within each group. Mann-Whitney *U* test was used for comparison between the differences of the data before and after exercise between the groups.

## Results

The general and clinical characteristics of the COPD patients are shown in Table 1. The profiles of the COPD patients are similar to those described in the literature, where patients are referred to pulmonary rehabilitation programs when the disease is in a moderate to severe stage. Peripheral oxygen saturation (SpO_2_) data of the patients before, during the peak, and after exercise are presented in Table 2.

**Table 1:** General and clinical characteristic of the COPD patients

Variables	COPD (*n* = 25)
Sex: male, n (%)	12 (48%)
Ethnicity: white, n (%)	20 (80%)
Age (years)^a^	64.40 ± 7.66
BMI (Kg/m^2^)^a^	24.76 ± 4.45
FEV_1_ (% predicted)	33.80 ± 14.84
FEV_1_/FVC (% predicted)	61.48 ± 19.22
COPD Status	
Moderate, n (%)	4 (16%)
Severe, n (%)	9 (36%)
Very Severe, n (%)	12 (48%)
Smoking Status	1/21/3
Never, n (%)	1 (4%)
Former, n (%)	21 (84%)
Current, n (%)	3 (12%)
Cigarettes (packs/year)^b^	
Current smokers	175 (35–525)
Former smokers	350 (35–1225)

*n* sample number, *%* frequency, *COPD* chronic obstructive pulmonary disease, *BMI* body mass index, *FEV*_*1*_ forced expiratory volume in 1 second, *FVC* forced vital capacity.

^a^Data are presented as mean and standard deviation;

^b^Median (minimum-maximum)

**Table 2:** Peripheral oxygen saturation (SpO_2_) data of the patients before, during the peak, and after exercise

Group	SpO_2_ at rest (before aerobic exercise)	SpO_2_ during the peak of exercise	SpO_2_ 20 min after exercise
AE group	94.0 ± 2.5%	87.0 ± 2.0%	94.04 ± 2.9%
AE-O_2_ group	94 ± 3.5%	90.0 ± 1.0%	94.4 ± 3.4%

Comet Assay results are shown in Table 3. For AE group, no significant observation in DI was found between pre- and post-exercise (*p* > 0.05), except for time zero (T0’) of MMS treatment (*p* = 0.01). Similarly, the same happened with T0’-DNA damage in the AE-O_2_ group (*p* = 0.01). These results can represent the heterogeneity of the experiments with humans. After the evaluation of the post-exercise situation between groups, some relevant findings were observed: AE group showed lower induced damage (1 h of MMS treatment) and consequently less DNA repair compared to AE-O_2_ group. AE-O_2_ group showed an increase in the induced DNA damage (1 h of MMS treatment) and an increased DNA repair capacity. Taking into consideration only the AE-O_2_ group, in the post-exercise situation the induced DNA damage after 1 h of MMS treatment was higher (*p* = 0.01) than in the pre-exercise.

**Table 3:** Results of the Comet Assay in COPD patients

Comet assay	AE group				AE-O_2_ group				p** between groups
	Pre-exercise	Post-exercise	p*	∆	Pre-exercise	Post-exercise	p*	∆	
Alkaline version	146.0 (92.5–209.5)	197.0 (94.5–974.0)	0.19	0 (−105.0–195.0)	248.0 (192.0–285.0)	302.0 (177.0–351.5)	0.20	11 (−137–169)	0.90
T0’ of MMS treatment	317.0 (277.0–366.5)	326.0 (239.0–370.5)	0.01	309.0 (193.0–392.0)	372.0 (30.5–394.5)	388.0 (339.0–398.0)	0.01	364.0 (168.0–392.0)	0.03
1 h of MMS treatment	317.0 (249.5–380.0)	296.0 (218.5–369.0)	0.18	−11.0 (−213.0–169.0)	336.0 (254.0–376.0)	368.0 (324.5–388.5)	0.01	17.0 (−40.0–283.0)	0.01
3 h of MMS treatment	259.0 (36.5–312.0)	240.0 (100.0–354.5)	0.19	4.0 (−52.0–114.0)	239.0 (154.0–295.0)	217.0 (163.0–271.0)	0.90	4.0 (−218.0–148.0)	0.37
Residual damage	62.9 (13.0–99.2)	81.6 (32.8–102.4)	0.51	1.7 (−97.1–36.3)	72.3 (48.2–94.0)	62.3 (49.8–85.5)	0.49	0.1 (−56.6–140.6)	0.33

Data are presented as Median (minimum-maximum);*Wilcoxon test; **Mann-Whitney *U* test; ∆: variation pre to post exercise; T0’: time zero of MMS treatment; *h* hours

## Discussion

Our results demonstrated that the aerobic exercise associated with O_2_ supplementation improved the DNA repair after MMS treatment. In contrast, COPD patients without O_2_ supplementation showed O_2_ desaturation during exercise and lower induced DNA damage and DNA repair. In this context, it is important to highlight that there is little evidence supporting the use of oxygen therapy to prevent or stimulate DNA repair in COPD patients [20]. For this reason, our results are quite relevant for the clinical management of COPD patients.

The inefficiency of DNA repair is a common finding in COPD individuals and it may be related to susceptibility for the development and progression of the disease [21]. In the hypoxic response, it has been shown that oxidative DNA damage in lungs of COPD patients is prominent [22]. It is known that the hypoxemia associated with COPD contributes to reduced quality of life, diminished exercise tolerance, skeletal muscle dysfunction, and ultimately increased risk of death. On the other hand, treatment of hypoxemia with oxygen therapy is one of the few interventions to extend life of hypoxemic COPD patients [6, 10, 23, 24].

In health subjects the physical exercise performed in normoxic conditions had no effect on the generation of DNA strand breaks, whereas hypoxic exercise produced more DNA strand breaks. This implies that during a hypoxic stress condition, the protection by the antioxidant system is insufficient to avoid generation of DNA strand breaks after exhaustive exercise [25]. Therefore, the prolonged exercise and high-intensity exercise in normoxic conditions may evoke inflammatory processes similar to hypoxia [25]. In addition, COPD patients have reduced muscle mass for many factors, including oxidative stress caused by increased reactive oxygen species, or systemic factors, such as inflammation, malnutrition, corticosteroid therapy, inactivity, smoking, aging, and hypoxemia. All these factors may contribute to muscle atrophy in COPD patients. These information suggest that an increase in damage/repair recurrence, possibly due to hypoxia, may exhaust the regenerative capacity of satellite cells in COPD patients with reduced muscle mass, leading to the senescence of these cells and muscle atrophy [20]. These results corroborate the findings in the AE group.

Pulmonary rehabilitation has been carefully evaluated in a large number of clinical trials. This treatment can increase peak workload, peak oxygen consumption, and endurance time in COPD patients [2]. These data are in agreement with those showing that regular exercise-induced adaptation attenuates the age-associated increased levels of oxidative stress in muscle cells. Additionally, it increases the activity of DNA repair, as well as the resistance against oxidative stress [26, 27].

There is evidence supporting the concept that hypoxia can drive and maintain genetic instability and a mutator phenotype. Genetic instability can arise as a function of hypoxia-mediated resistance to apoptosis and decreased DNA repair, leading to increased rates of mutagenesis and modifications in chromatin. This might be particularly true in proliferating cells that have adapted to low O_2_ levels and continue to proliferate in the context of compromised DNA repair [28]. This information can help to explain the fact that in the AE group there was less damage induction and DNA repair. According to Radak et al. [27], this possibly represents an adaptive response of the cell to these molecular insults.

## Conclusion

We conclude that COPD patients who exercise with oxygen supplementation have an increased response to DNA damage induced by MMS. In addition, they have a better DNA repair when compared to physical exercise condition without oxygen supplementation.
